# The correlation between serum uric acid and diabetic kidney disease in adult-onset type 1 diabetes patients in China

**DOI:** 10.1007/s00592-023-02119-7

**Published:** 2023-06-01

**Authors:** Jun Jiang, Xiaowan Zhou, Lei Lan, Jianping Weng, Wei Ren

**Affiliations:** 1 Cheeloo College of Medicine, Shandong University, Jinan, 250021 Shandong China; 2 The Department of Nephrology, The First Affiliated Hospital of USTC, Division of Life Sciences and Medicine, University of Science and Technology of China, Hefei, 230001 Anhui China; 3 The Department of Endocrinology, The First Affiliated Hospital of USTC, Division of Life Sciences and Medicine, University of Science and Technology of China, Hefei, 230001 Anhui China

**Keywords:** Adult-onset, Type 1 diabetes mellitus, Diabetic kidney disease, Serum uric acid

## Abstract

**Background/aim:**

To assess the correlation between serum uric acid (UA) level and diabetic kidney disease among adult-onset Type 1 diabetes mellitus (T1DM) patients in China.

**Methods:**

A total of 184 patients with adult-onset T1DM between January 2014 and December 2016 were recruited, with demographics and medical data collected. Comparisons were performed between according to different serum UA gender-specific quartiles. Relationship between serum UA level with urinary ACR and eGFR was also assessed.

**Results:**

Median urinary ACR and eGFR were 21.55 [10.79, 45.02] mg/g and 113.86 [88.43, 143.61] ml/min/1.73 m^2^, respectively. The median UA was 257.4 (208.2–334.8) μmol/L. Participants with higher serum UA levels had higher urinary ACR and lower eGFR than those with lower UA (*P* < 0.05). Higher serum UA level was significantly associated with higher urinary ACR in Spearman's correlational analysis (*P* = 0.006) and multiple stepwise regression analysis (*P* = 0.013). The association between serum UA and urinary ACR was not linear, but showed a curve correlation, which also showed in the sensitivity analysis. Serum UA in the upper gender-specific quartile, was associated with lower eGFR (*P* < 0.001) and showed an independent negative correlation with eGFR in multiple stepwise regression analysis (*P* < 0.001).

**Conclusions:**

The serum UA level was negatively correlated with eGFR and had a curve correlation with urinary ACR in adult-onset T1DM patients of China.

**Supplementary Information:**

The online version contains supplementary material available at 10.1007/s00592-023-02119-7.

## Introduction

Diabetic kidney disease (DKD) is one of the most common chronic kidney diseases (CKD), closely related to end-stage renal disease (ESRD) and increased risk of cardiovascular disease (CVD) in type 1 diabetes mellitus (T1DM) [1–3]. Most newly diagnosed T1DM patients in China were adult-onset, while in European and America were adolescent [4], and the previous evidence had shown that characteristics of DKD could be heterogeneous among patients from different ethnicities [5–7]. It is essential to explore the modifiable factors of DKD for delaying the progression of DKD in adult-onset T1DM patients in China.

Hyperuricemia is one of the modifiable factors, contributing to cardiovascular disease, CKD, and hypertension [8–10]. The crude and age-standardized prevalence of hyperuricemia was 10.24% and 12.60%, respectively, in the Chinese rural population [11], while the prevalence in the USA was over 20% [12]. Several studies had concentrated on the relationship between serum uric acid (UA) levels and DKD in adolescent-onset T1DM patients, but the conclusions are inconsistent [13–19]. Some studies have indicated that elevated serum UA levels were strong and independent predictors of albuminuria and early glomerular filtration rate (GFR) decline in T1DM patients [13–17], and the others revealed no causal effects of the serum UA on the estimated GFR or the risk of CKD in T1DM patients [18, 19]. So far, evidence regarding on the association between DKD and serum UA in adult-onset T1DM patients, particularly in Asian populations, was still scarce. Given the heterogeneity of DKD among ethnicities, inconsistent research results of previous studies, and no research focused on the association between serum UA and DKD in adult-onset T1DM patients, in this study, we conducted a cross-sectional study to analyze the association between serum UA level and DKD in adult-onset T1DM patients of China.

## Methods

### Patients

This study was a cross-sectional study conducted in a single medical center in Anhui, China. A total of 184 hospitalized patients were recruited who were considered to have adult-onset T1DM presenting at the Department of Endocrinology in Anhui Provincial Hospital between January 2014 and December 2016. The patients were excluded if they had the following reasons: adolescent-onset T1DM, no data of the serum UA level, documented ketosis or ketoacidosis in the 3 months before enrollment, documented glomerulonephritis, systemic diseases, heart failure, urinary tract infections, or active gout. The patients, especially with DKD in our study, had not received high-protein diet and health education about a high-protein diet at enrollment. The research ethics committee of Anhui Provincial Hospital approved the study design (NO.2022-RE-331).

### Clinical and laboratory measurements

Details of methodology of research and data collection were described in our previous study [20]. Demographic and clinical data were retrospectively collected via the hospital's database. During clinical practice, patients in our study who previously had at least 3 consecutive ACR monitoring would provide only one ACR test during hospitalization, while those without or less would be provided 3 consecutive ACR monitoring during hospitalization. When analyzing the correlation between ACR and serum UA, ACR performed on the same day as serum UA detection was selected for analysis.

### Definitions

Hyperuricaemia was typically reported when serum UA was higher or equal to 420umol/L (7 mg/dl) [9]. The clinical diagnosis of T1DM was based on American Diabetes Association's descriptions of T1DM [21]. The adult-onset T1DM refers to the onset of T1DM aged 18 years or older. According to Standards of Medical Care in Diabetes-2022 of the American Diabetes Association, DKD was diagnosed [22]. According to the guideline of Kidney Disease: Improving Global Outcomes (KDIGO), the progression risk of DKD was classified, which included the GFR category (G1–G5) and albuminuria category (A1–A3) [23]. Urine albumin excretions were assessed by urinary ACR in spot urine samples. Normoalbuminuria (A1) was defined as a urinary ACR of less than 30 mg/g, while microalbuminuria (A2) had been previously recorded or was a urinary ACR between 30 and 299 mg/g, and macroalbuminuria (A3) had been previously recorded or was a urinary ACR greater than 300 mg/g in two of three consecutive measurements [22].

### Statistical analysis

All statistical analyses were performed using IBM SPSS Statistics 20.0 version (IBM Corporation, Armonk, NY, USA). A normal distribution of continuous variables was summarized as means ± standard deviation (SD), while a skewed distribution of continuous variables was expressed as medians with interquartile ranges. For continuous data with a skewed distribution among patients in gender-specific quartiles of serum UA, nonparametric tests were used for statistical analyses. The number (n) and percentage (%) in each category were calculated for categorical variables. The categorical variables were evaluated with a *Chi-square* test or *Fisher* exact test. The relationship between serum UA level and urinary ACR was performed by Spearman’s correlational analysis, Pearson's correlational analysis, and multiple stepwise regression analysis. A *P* value < 0.05 was considered statistically significant.

## Results

### Patient characteristics and comparison of baseline covariates

**Fig. 1:**
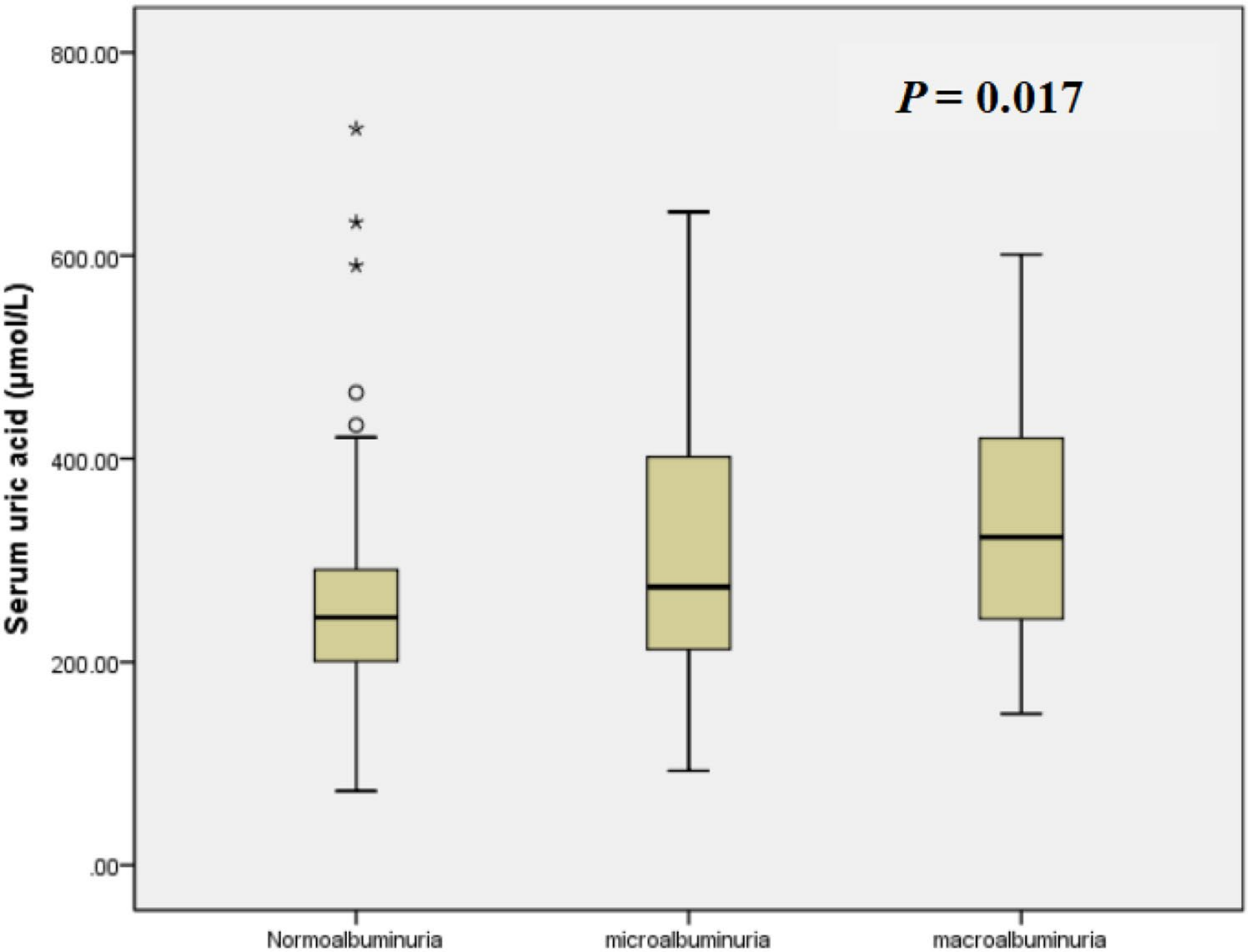
Serum UA in groups of albuminuria. Data represent median (*P*25, *P*75). Participants were categorized as normoalbuminuria (ACR < 30 mg/g), microalbuminuria (ACR 30–299 mg/g), and macroalbuminuria (ACR > 300 mg/g)

Of 184 adult-onset T1DM patients, 98 (53.3%) were male with median onset age of 29 [23, 36] years and median diabetic duration of 5.0 [1.0, 10.0] years. The median urinary ACR and eGFR were 21.55 [10.79, 45.02] mg/g and 113.86 [88.43, 143.61] ml/min/1.73 m^2^, respectively. The number of patients with G1, G2, G3a, G3b, G4, and G5 stages was 135, 29, 9, 6, 2, and 3, respectively, while with the A1, A2, and A3 stages was 118, 44, and 22, respectively. The patients with low DKD progression risk, moderately increased DKD progression, high DKD progression risk, and very high DKD progression risk were 116, 42, 8, and 18, respectively. The median UA was 257.4 (208.2–334.8) μmol/L, and the median dosage of insulin performed by patients was 0.59 [0.43, 0.78] U/kg/d. The baseline characteristics of the 184 adult-onset T1DM patients divided according to the gender-specific quartiles of serum UA are shown in Table 1. Participants with higher UA had longer diabetes duration, higher TC and urinary ACR, and lower eGFR than those with lower UA (*P* < 0.05 for all). However, there was no significant difference among all quartiles of serum UA in onset age, BMI, HbA1c, SBP, DBP, Hb, TG, HDL-c, LDL-c, ALB, the proportion of beta-blockers, loop diuretics, angiotensin-converting enzyme (ACE) inhibitors, lowering serum uric acid drug treatment, or the dosage of insulin performed by patients. Figure 1 and Figure Supplement 1 illustrate the median values of serum UA according to the albuminuria group. Serum UA level was significantly higher with increasing levels of albuminuria (*P* < 0.05).

**Table 1:** Clinical characteristics divided according to the gender-specific quartiles of serum UA

Characteristics Serum UA (μmol/L)	Female: UA < 167.8 Male: UA < 238.3 n = 45	Female: 167.8 ≤ UA < 231.5 Male: 238.3 ≤ UA < 272.0 *n* = 47	Female: 231.5 ≤ UA < 303.5 Male: 272.0 ≤ UA < 357.2 *n* = 47	Female: UA ≥ 303.5 Male: UA ≥ 357.2 *n* = 45	*P* value
Male/female	24/21	25/22	25/22	24/21	1.00
Age of onset (years)	31.5 [25.0, 40.5]	29.0 [25.0, 36.0]	27.0 [23.0, 36.0]	26.0 [21.0, 34.0]	0.11
Duration of diabetes (years)	2.0 [0.5, 8.0]	5.0 [1.0, 8.0]	4.0 [1.0, 8.0]	8.0 [3.5, 11.5]	**0.007**
BMI (kg/m^2^)	20.4 [18.1, 21.6]	19.6 [18.3, 22.6]	19.9 [17.7, 21.6]	19.5 [18.1, 22.1]	0.995
HbA1c (%)	9.6 [8.1, 11.2]	8.3 [7.4, 11.2]	8.5 [7.5, 10.8]	9.5 [6.8, 12.5]	0.28
SBP (mmHg)	120.0 [110.0, 130.0]	120.0 [110.0, 136.0]	120.0 [110.0, 130.0]	123.0 [110.0, 133.5]	0.67
DBP (mmHg)	80.0 [71.0, 83.0]	76.0 [70.0, 86.0]	80.0 [71.0, 85.0]	80.0 [70.0, 85.0]	0.88
Hb (g/L)	128.0 [119.0, 137.0]	127.0 [118.0, 141.0]	134.0 [120.0, 141.0]	125.0 [107.5, 140.5]	0.27
TC (mmol/L)	3.87 [3.44, 4.43]	4.34 [3.72, 4.85]	4.26 [3.79, 4.94]	4.49 [3.79, 5.03]	**0.023**
TG (mmol/L)	0.78 [0.63, 1.23]	0.89 [0.61, 1.09]	0.90 [0.64, 1.35]	1.05 [0.90, 1.82]	0.24
HDL-C (mmol/L)	1.21 [090, 1.39]	1.36 [1.09, 1.74]	1.09 [0.93, 1.51]	1.07 [0.92, 1.46]	0.17
LDL-c (mmol/L)	2.09 [1.67, 2.53]	2.29 [1.96, 2.87]	2.22 [1.81, 2.89]	2.62 [1.95, 3.02]	0.06
ALE (g/L)	38.8 [36.7, 41.0]	38.4 [35.3, 42.6]	39.4 [37.6, 41.4]	39.9 [34.7, 43.2]	0.21
eGFR (ml/min/1.73m^2^)	132.4 [110.6, 172.1]	117.6 [101.2, 148.0]	117.0 [90.9, 144.2]	71.5 [45.7, 104.8]	** < 0.001**
Urinary ACR (mg/g)	19.86 [8.98, 29.45]	14.97 [9.21, 27.62]	22.78 [10.39, 39.52]	42.77 [17.93, 434.63]	**0.017**
*Antihypertensive drug treatment*					
Beta-blockers [*n* (%)]	0 (0%)	0 (0%)	1 (2.13%)	1 (2.22%)	0.365
Loop diuretics [*n* (%)]	0 (0%)	0 (0%)	0 (0%)	1 (2.22%)	0.241
ACE inhibitors [*n* (%)]	0 (0%)	1 (2.13%)	3 (6.38%)	1 (2.22%)	0.260
Drugs lowering serum UA [*n* (%)]	0 (0%)	0 (0%)	0 (0%)	0 (0%)	^–^
Insulin performed by patients (U/kg/d)	0.56 [0.49, 0.89]	0.59 [0.42, 0.84]	0.56 [0.44, 0.77]	0.60 [0.44, 0.68]	0.939

Bold values are statistically significant

*ACR* albumin–creatinine ratio, *ALB* albumin, *BMI* body mass index, *Cr* creatinine, *DBP* diastolic blood pressure, *eGFR* estimated glomerular filtration rate, *Hb* hemoglobin, *HbA1c* glycated hemoglobin A1c, *HDL-C* high-density lipoprotein cholesterol, *LDL-C* low-density lipoprotein cholesterol, *SBP* systolic blood pressure, *TC* total cholesterol, *TG* triglyceride, *UA* uric acid, *ACE* angiotensin-converting enzyme

### Correlations between serum UA levels and albuminuria

**Fig. 2:**
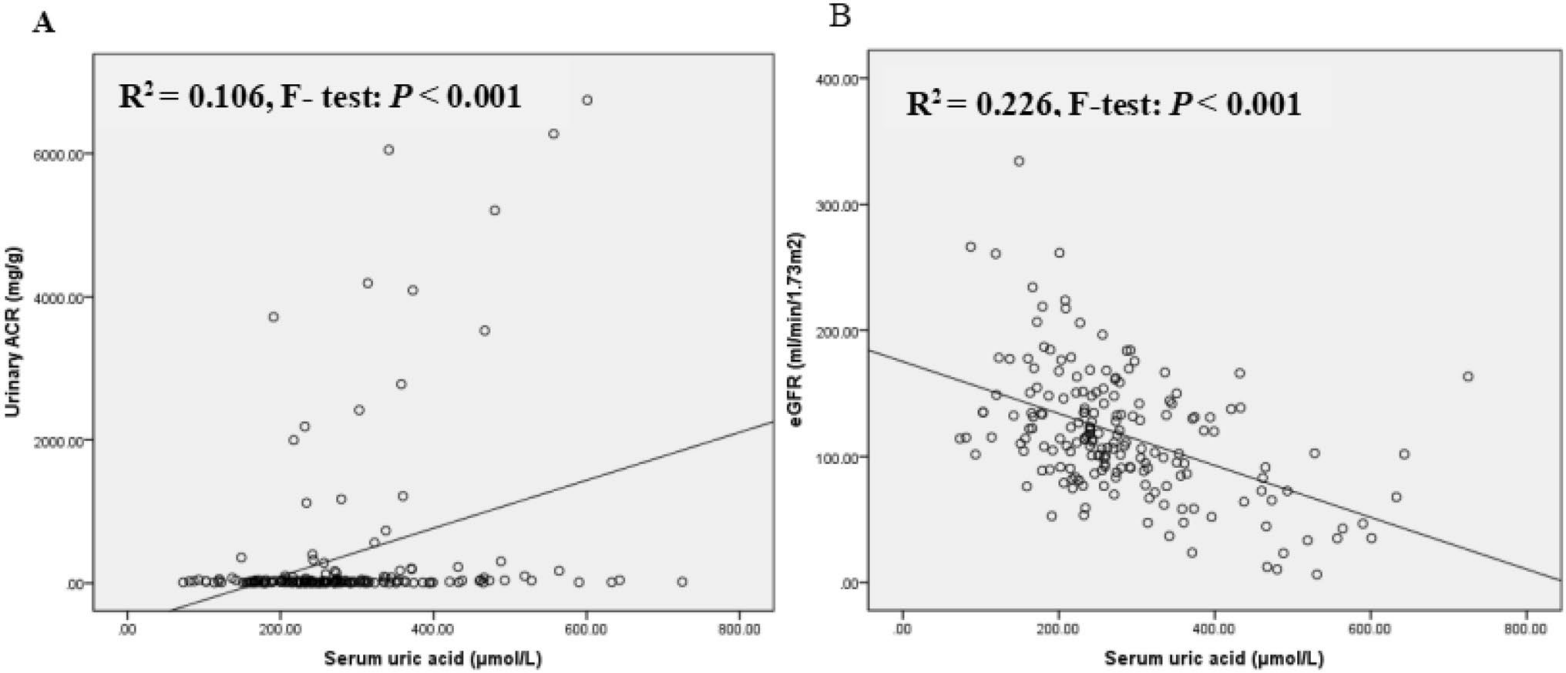
Correlations with serum UA and **A** urinary ACR and **B** eGFR

**Fig. 3:**
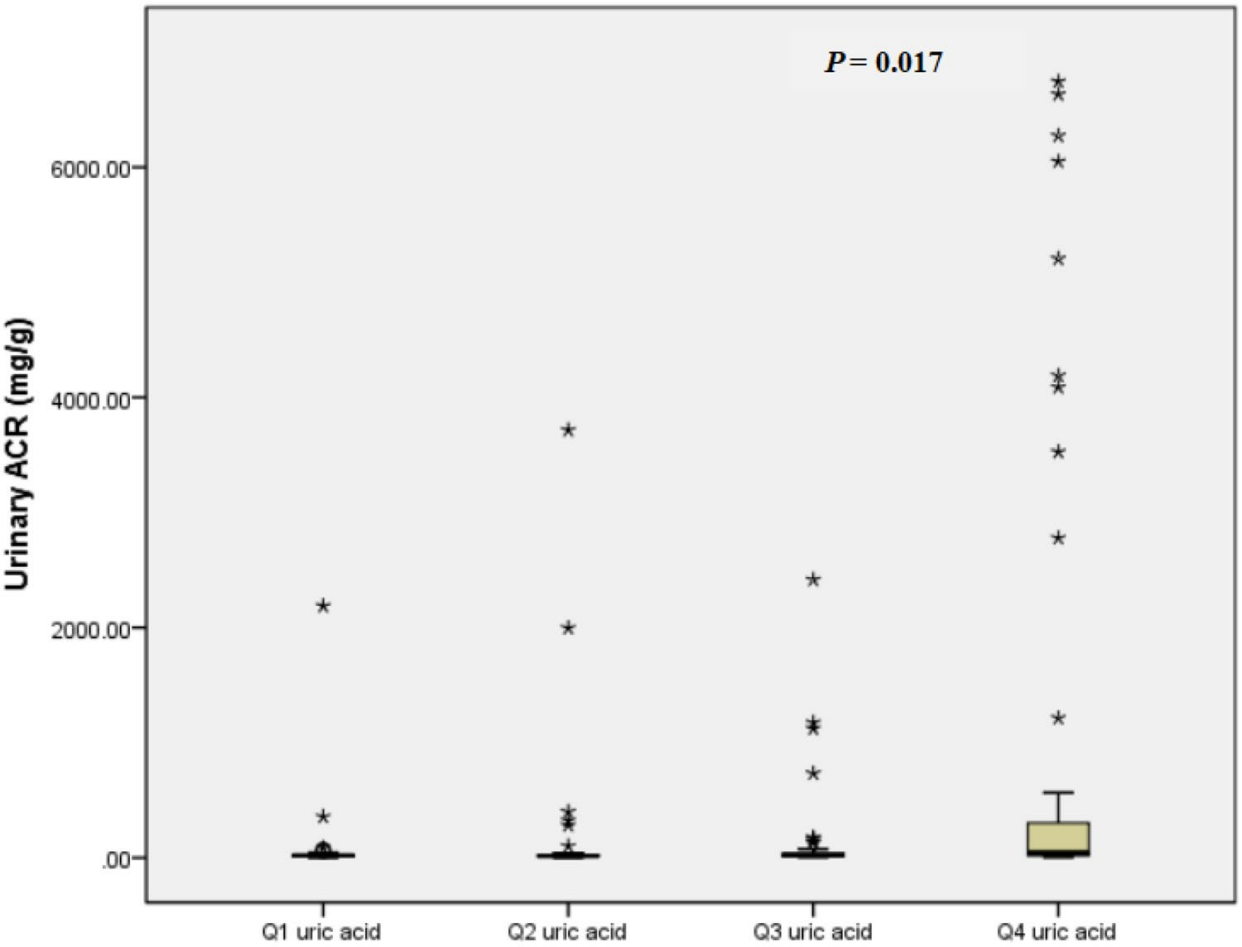
Urinary ACR in gender-specific quartiles of serum uric acid. Data represent the median (*P*25, *P*75)

To analyze the correlations between serum UA levels and albuminuria, Spearman's correlation analysis was conducted to examine the factors associated with serum UA. Table 2 shows that serum UA level is positively correlated with the duration of DM, serum TG concentrations, and urinary ACR and negatively associated with eGFR (all* P* < 0.05). No significant correlation was observed between serum UA level and HbA1c as well as insulin dosage performed by patients. After adjustment for those significant factors, stepwise multiple linear regression analysis showed an independent positive association between serum UA levels and urinary ACR and a negative correlation between serum UA levels and eGFR (*P* < 0.05). Higher UA was significantly associated with higher urinary ACR in unadjusted (Fig. 2A: *R*^2^ = 0.106, *F*-test: *P* < 0.0001) (Fig. 3, *P* = 0.017) (Figure Supplement 2, *P* = 0.007) and multiple stepwise regression analysis (*P* = 0.013). Although the *P* value was significant in Fig. 2A, the *R*-value was low. The association between serum uric acid and urinary ACR was nonlinear but showed a curve correlation (Figure Supplement 2), in both female and male T1DM patients (Figure Supplement 3).

**Table 2:** The correlated factors with serum UA levels

Parameter	Spearman’s correlation analysis		Stepwise multiple linear regression	
	*r*	*P* value	*β* coefficient ± SE	*P* value
Age of onset (years)	− 0.139	0.06		
Duration of diabetes (years)	0.273	** < 0.001**		
BMI (kg/m2)	− 0.037	0.64		
HbA1c (%)	− 0.086	0.25		
SBP (mmHg)	0.05	0.50		
DBP (mmHg)	0.055	0.46		
Hb (g/L)	0.123	0.10		
TC (mmol/L)	0.108	0.16		
TG (mmol/L)	0.216	**0.005**	17.93 ± 7.3	0.015
HDL-C (mmol/L)	− 0.102	0.19		
LDL-C (mmol/L)	0.116	0.14		
ALB (g/L)	0.057	0.44		
Urinary ACR (mg/g)	0.204	**0.006**	0.016 ± 0.007	0.013
eGFR (ml/min/1.73m^2^)	− 0.455	** < 0.001**	− 0.882 ± 0.168	< 0.001
The dosage of insulin performed by patients (U/kg/d)	− 0.08	0.403		

Underlined values indicate the changes in my revised version

*ACR* albumin–creatinine ratio, *SE* standard error, *ALB* albumin, *BMI* body mass index, *DBP* diastolic blood pressure, *eGFR* estimated glomerular filtration rate, *Hb* hemoglobin, *HbA1c* glycated hemoglobin A1c, *HDL-C* high-density lipoprotein cholesterol, *LDL-C* low-density lipoprotein cholesterol, *SBP* systolic blood pressure, *TC* total cholesterol, *TG* triglyceride, *UA* uric acid

### Association between serum UA levels and eGFR

**Fig. 4:**
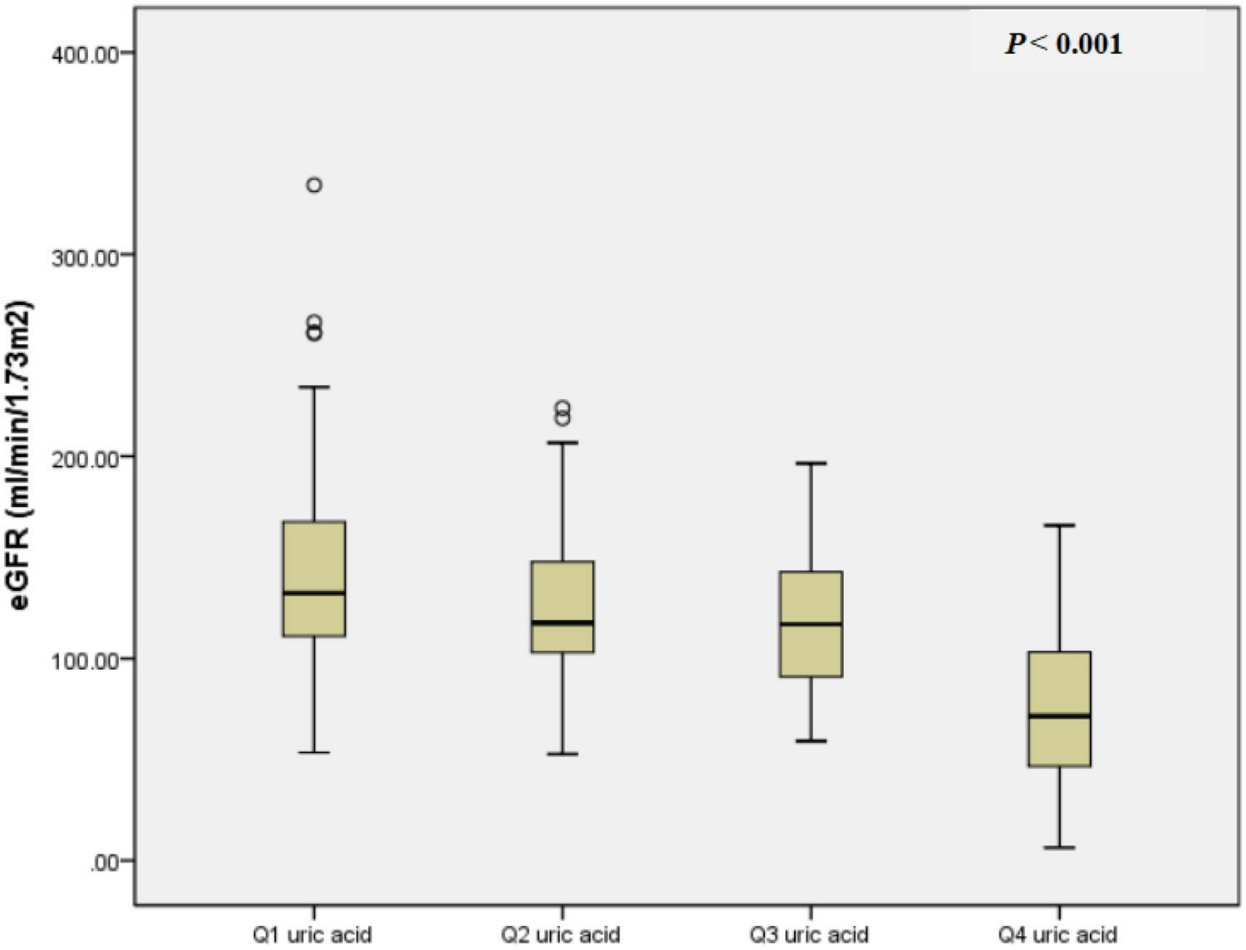
eGFR in gender-specific quartiles of serum uric acid. Data represent the median (*P*25, *P*75)

UA in the upper gender-specific quartile was associated with lower eGFR (Table 2; Figs. 2B and 4) (*P* < 0.05 in all), and multiple stepwise regression analysis also showed that an independent negative correlation between serum UA levels and eGFR (Table 2, *P* < 0.001). Figure Supplement 4 also shows that UA in the upper quartile was associated with lower eGFR in both female and male T1DM patients (*P* < 0.05 in both).

To further analyze the relationship between the serum UA level with ACR and eGFR, Pearson's correlation analysis was conducted according to the classification of DKD progression risk (Table Supplement 1). Serum UA and urinary ACR had a curve correlation, which showed a positive linear correlation at macroalbuminuria and no linear correlation at less than macroalbuminuria (Table Supplement 1). Serum UA level was negatively correlated with eGFR no matter whether eGFR was normal or decreased, and the correlation between UA and eGFR gradually increased with the DKD progression risk. Serum UA was also observed to have a negative correlation with eGFR in patients who had a value of HbA1c less than 7.0%. Furthermore, serum UA had no correlation with urinary ACR in patients with HbA1c less than 7.0%, which was associated with a lower likelihood of macroalbuminuria in patients with good glycemic control (Table Supplement 2).

## Discussion

DKD is one of the most common chronic kidney diseases (CKD) and remains the leading cause of morbidity and mortality in T1DM patients. Evidence has proved that the urinary albumin levels in T1DM patients may progressively increase five years after diagnosis [24]. Microalbuminuria can predict the progression of DKD in T1DM patients [25]. Still, no evidence focused on the association between serum UA and DKD in adult-onset T1DM patients, especially in China. In this present cross-sectional research, we first discovered that the serum UA level was negatively correlated with eGFR and had a curve correlation with urinary ACR in adult-onset T1DM patients in China. Serum UA and urinary ACR had a positive linear correlation at macroalbuminuria and no linear correlation at less than macroalbuminuria.

Previous studies revealed that high serum UA might contribute to initiating arterial hypertension while it has little effect on already-established hypertension [26]. Several animals research revealed that hyperuricemia promotes the development of hypertension to accelerate renal function deterioration [27, 28]. We speculated that early phases of hyperuricemia contributed to hypertension. This association gradually weakened to disappear even though the hyperuricemia persisted. This is partially explained by the fact that the serum UA level did not correlate with blood pressure, neither SBP nor DBP in our study.

Although hyperuricemia was not correlated with hypertension, it was positively correlated with high TG levels in this research (Table 2). This article also found that participants with higher UA had higher serum TC levels, while the BMI, serum ALB, HbA1c, the proportion of beta-blockers, loop diuretics, angiotensin-converting enzyme (ACE) inhibitors, lowering serum uric acid drug treatment, and the dosage of insulin requirement had no significant difference across the quartiles of serum UA (Table 1). These results suggested that the correlation between serum UA and hyperlipemia may be independent of patients' blood glucose levels and nutritional status. As we all know, hyperlipemia is an independent risk factor for DKD in T1DM patients and cardiovascular disease (CVD) [29, 30]. This is easy to understand that hyperuricemia is related to CVD and DKD in previous studies [8–10]. Our study also confirmed that serum UA was correlated with urinary ACR and negatively with eGFR. The effect and mechanism of TG and serum UA on DKD in T1DM patients are worthy of further investigation.

However, the Preventing Early Renal Loss in Diabetes (PERL) trial showed no significant benefits of serum UA reduction with allopurinol on kidney outcomes among patients with T1DM and early-to-moderate DKD, in which the included patients had a long course of disease (the mean age 51.1 years, the mean duration of diabetes 34.6 years) and the renal complications (most eGFR < 90 ml/min/1.73m^2^) [31].

These findings cannot explain the association between serum UA levels and DKD. In addition, the FEATHER trial found that stage 3 CKD patients with hyperuricemia, who had no proteinuria and a higher baseline renal function, got significant benefits in renal outcomes from lowering Serum UA [32]. For timely interventions in the early phases of hyperuricemia may prove more beneficial than treatment at later stages, the PERL study may miss the best opportunity for intervention [8, 26]. And trials enrolling patients at an earlier stage of T1DM with hyperuricemia could lead to different conclusions from the PERL studies. Furthermore, the majority of patients included in the PERL study were adolescent-onset T1DM patients. The findings may not be applied to adult-onset T1DM patients in China.

Some research has revealed the possible mechanism of serum UA levels on DKD. First, UA may directly cause renal inflammation promoting intrarenal inflammation, interstitial fibrosis, albuminuria, and chronic kidney disease development by depositing intraluminal micro-crystals in collecting ducts [33–36]. Additionally, several animal research revealed that hyperuricemia promoted the development of hypertension to accelerate renal function deterioration [27, 28]. Hyperuricemia could accelerate renal function deterioration via high systemic blood pressure and cyclooxygenase-mediated, thromboxane-induced vascular disease [37]. Experimental studies demonstrated high serum uric acid levels promoted medial thickening of preglomerular arterioles and were directly correlated with glomerular capillary pressure [38], which led to ischemia and hypoxia, and tubulointerstitial fibrosis [39]. Lytvyn et al*.* suggested that plasma UA-mediated afferent arteriolar resistances of patients with T1DM may be caused by the thickening of the afferent renal arteriole, potentiating renal injury by causing renal microcirculation ischemia [40]. Lytvyn et al*.* also found that UA lowering in patients with T1DM lowered systolic BP and modulated the renal efferent resistance responses to hyperglycemia but without impacting the RAAS or NO levels, suggesting that plasma UA may augment other hemodynamic or inflammatory mechanisms that control the renal response to hyperglycemia at the efferent arteriole [41]. Furthermore, serum UA levels in the normal range could decrease endothelium-dependent reactions associated with T1DM, and serum UA levels were associated with microvascular endothelial dysfunction in patients with T1DM [42]. Finally, hyperuricemia could increase the risk of segmental glomerulosclerosis, tubular atrophy, and interstitial fibrosis [43].

However, this research had several limitations. First, our study was a single-center, cross-sectional observational research. We could not get a causal relationship between the serum UA and DKD. No follow-up data was also a limitation as we cannot further analyze the relationship between serum UA and DKD progression. Second, our study did not analyze the relationship between the trend of serum UA level with DKD and the mechanism that serum UA on DKD. Third, whether our research findings suit other groups remains uncertain. The effect and mechanism of TG and hyperuricemia on DKD in adult-onset T1DM patients are worthy of further investigation.

## Conclusion

The serum UA level was negatively correlated with eGFR and had a curve correlation with urinary ACR in adult-onset T1DM patients in China.

## Supplementary Information

Below is the link to the electronic supplementary material.

Supplementary file1 (DOC 130 kb)

## References

[1] Wadén J, Forsblom C, Thorn LM (2009). Adult stature and diabetes complications in patients with type 1 diabetes: the FinnDiane Study and the diabetes control and complications trial. Diabetes.

[2] Tonelli M, Muntner P, Lloyd A (2012). Risk of coronary events in people with chronic kidney disease compared with those with diabetes: a population-level cohort study. Lancet.

[3] Diabetes Prevention Program Research Group (2009). Changes in albumin excretion in the diabetes prevention program. Diabetes Care.

[4] Weng J, Zhou Z, Guo L (2018). Incidence of type 1 diabetes in China, 2010–13: population based study. BMJ.

[5] Wang Y, Tan J, Liu D (2019). The association of UNC13B gene polymorphisms and diabetic kidney disease in a Chinese Han population. Med Sci Monit.

[6] Jin L, Wang T, Jiang S (2017). The association of a genetic variant in *SCAF8-NKSR3* with diabetic kidney disease and diabetic retinopathy in a Chinese population. J Diabet Res.

[7] Jenny PD, Michelle S, Sherita HG (2019). Racial/ethnic trends in prevalence of diabetic kidney disease in the United States. Kidney Int Rep.

[8] Ponticelli C, Podestà MA, Moroni G (2020). Hyperuricemia as a trigger of immune response in hypertension and chronic kidney disease. Kidney Int.

[9] Dalbeth N, Gosling AL, Gaffo A, Abhishek A (2021). Gout. Lancet.

[10] Dehlin M, Jacobsson L, Roddy E (2020). Global epidemiology of gout: prevalence, incidence, treatment patterns and risk factors. Nat Rev Rheumatol.

[11] Dong X, Zhang H, Wang F, Liu X, Yang K, Tu R, Wei M, Wang L, Mao Z, Zhang G, Wang C (2020). Epidemiology and prevalence of Hyperuricemia among men and women in Chinese rural population: the Henan Rural Cohort Study. Mod Rheumatol.

[12] Chen-Xu M, Yokose C, Rai SK, Pillinger MH, Choi HK (2019). Contemporary prevalence of gout and hyperuricemia in the United States and decadal trends: the National Health and Nutrition Examination Survey, 2007–2016. Arthritis Rheumatol.

[13] Hovind P, Rossing P, Tarnow L, Johnson RJ, Parving H-H (2009). Serum uric acid as a predictor for development of diabetic nephropathy in type 1 diabetes: an inception cohort study. Diabetes.

[14] Jalal DI, Rivard CJ, Johnson RJ (2010). Serum uric acid levels predict the development of albuminuria over 6 years in patients with type 1 diabetes: findings from the Coronary Artery Calcification in type 1 diabetes study. Nephrol Dial Transplant.

[15] Ficociello LH, Rosolowsky ET, Niewczas MA (2010). High-normal Serum uric acid increases risk of early progressive renal function loss in type 1 diabetes: results of a 6-year follow-up. Diabetes Care.

[16] Pilemann-Lyberg S, Lindhardt M, Persson F, Andersen S, Rossing P (2018). Serum uric acid and progression of diabetic nephropathy in type 1 diabetes. J Diabet Complic.

[17] Mauer M, Doria A (2018). Uric acid and diabetic nephropathy risk. Contrib Nephrol.

[18] Ahola AJ, Sandholm N, Forsblom C (2017). The serum uric acid concentration is not causally linked to diabetic nephropathy in type 1 diabetes. Kidney Int.

[19] Pilemann-Lyberg S, Hansen TW, Persson F (2018). Uric acid is not associated with diabetic nephropathy and other complications in type 1 diabetes. Nephrol Dial Transplant.

[20] Jiang J, Lan L, Zhou X, Peng L, Ren W (2018). The relationship between haemoglobin level and type 1 diabetic nephropathy in Han patients in Anhui. China Intern Med J.

[21] American Diabetes Association Professional Practice Committee; 2. Classification and diagnosis of diabetes: standards of medical care in diabetes-2022. Diabetes Care 2022, 45(Supplement-1): S17–S38. 10.2337/dc22-S002 10.2337/dc22-S002 34964875

[22] American Diabetes Association Professional Practice Committee; 11. Chronic Kidney Disease and Risk Management: Standards of Medical Care in Diabetes-2022. Diabetes Care, 2022; 45(Supplement_1): S175–S184. 10.2337/dc22-S011. 10.2337/dc22-S011 34964873

[23] Kidney Disease: Improving Global Outcomes (KDIGO) Diabetes Work Group. KDIGO 2022 Clinical Practice Guideline for Diabetes Management in Chronic Kidney Disease. Kidney Int, 2022;102(5S):S1–S127. 10.1016/j.kint.2022.06.008. 10.1016/j.kint.2022.06.008 36272764

[24] Molitch ME, DeFronzo RA, Franz MJ (2004). Nephropathy in diabetes. Diabetes Care.

[25] Mogensen CE, Christensen CK (1984). Predicting diabetic nephropathy in insulin-dependent patients. N Engl J Med.

[26] Feig DI, Soletsky B, Johnson RJ (2008). Effect of allopurinol on blood pressure of adolescents with newly diagnosed essential hypertension: a randomized trial. JAMA.

[27] Mazzali M, Kanellis J, Han L (2002). Hyperuricemia induces a primary renal arteriolopathy in rats by a blood pressure-independent mechanism. Am J Physiol Renal Physiol.

[28] Sánchez-Lozada LG, Tapia E, Santamaría J (2005). Mild Hyperuricemia induces vasoconstriction and maintains glomerular hypertension in normal and remnant kidney rats. Kidney Int.

[29] Perkins BA, Bebu I, de Boer IH (2019). Risk factors for kidney disease in type 1 diabetes. Diabetes Care.

[30] Tsion A, Eric DP, Neha JP (2020). The association between triglycerides and incident cardiovascular disease: What is “Optimal”?. J Clin Lipidol.

[31] Doria A, Galecki AT, Spino C (2020). Serum Urate Lowering with Allopurinol and Kidney Function in Type 1 Diabetes. N Engl J Med.

[32] Kimura K, Hosoya T, Uchida S (2018). Febuxostat therapy for patients with stage 3 CKD and asymptomatic hyperuricemia: a randomized trial. Am J Kidney Dis.

[33] Yang Z, Xiaohua W, Lei J (2010). Uric acid increases fibronectin synthesis through upregulation of lysyl oxidase expression in rat renal tubular epithelial cells. Am J Physiol Renal Physiol.

[34] Crișan TO, Cleophas MCP, Oosting M (2016). Soluble uric acid primes TLR-induced proinflammatory cytokine production by human primary cells via inhibition of IL-1Ra. Ann Rheum Dis.

[35] Xiao J, Fu C, Zhang X (2015). Soluble monosodium urate, but not its crystal, induces toll like receptor 4-dependent immune activation in renal mesangial cells. Mol Immunol.

[36] Ryu E-S, Kim MJ, Shin H-S (2013). Uric acid-induced phenotypic transition of renal tubular cells as a novel mechanism of chronic kidney disease. Am J Physiol Renal Physiol.

[37] Kang D-H, Nakagawa T, Feng L (2002). A role for uric acid in the progression of renal disease. J Am Soc Nephrol.

[38] Sánchez-Lozada LG, Tapia E, Avila-Casado C (2002). Mild hyperuricemia induces glomerular hypertension in normal rats. Am J Physiol Renal Physiol.

[39] Liu M, Ning X, Li R (2017). Signalling pathways involved in hypoxia-induced renal fibrosis. Cell Mol Med.

[40] Lytvyn Y, Škrti M, Yang GK (2016). Plasma uric acid effects on glomerular hemodynamic profile of patients with uncomplicated Type 1 diabetes mellitus. Diabet Med.

[41] Lytvyn Y, Har R, Locke A (2017). Renal and vascular effects of uric acid lowering in normouricemic patients with uncomplicated type 1 diabetes. Diabetes.

[42] Matheus A, Tibiriçá E, da Silva P (2011). Uric acid levels are associated with microvascular endothelial dysfunction in patients with Type 1 diabetes. Diabet Med.

[43] Fan S, Zhang P, Wang AY (2019). Hyperuricemia and its related histopathological features on renal biopsy. BMC Nephrol.

