# Supplementary material for: The correlation between serum uric acid and diabetic kidney disease in adult-onset type 1 diabetes patients in China
Source: Acta Diabetol. 2023 Jun 1;60(9):1231–9. doi: 10.1007/s00592-023-02119-7 (PMC10359385; doi:10.1007/s00592-023-02119-7)
Supplement: Supplementary file 1 — Supplementary file1 (DOC 130 kb) [file 592_2023_2119_MOESM1_ESM.doc]

**Table Supplement 1.** **Pearson's correlations between serum UA with urinary ACR and eGFR according to** **the classification of DKD** progression risk

| Classification | Urinary ACR | | eGFR | |
| --- | --- | --- | --- | --- |
|  | R | *P* – value | R | *P* - value |
| All patients (n = 184) | **0.325** | **<0.001** | **- 0.475** | **<0.001** |
| Low DKD progression risk patients  (n = 116) | - 0.10 | 0.287 | **- 0.213** | **0.022** |
| Moderately increased DKD progression risk patients (n = 42) | - 0.037 | 0.818 | **- 0.556** | **< 0.001** |
| High and very high DKD progression risk patients (n = 26) | 0.360 | 0.07 | **- 0.601** | **0.001** |
| A1 (n = 118) | - 0.106 | 0.252 | **- 0.274** | **0.003** |
| A2 (n = 44) | 0.143 | 0.354 | **- 0.593** | **< 0.001** |
| A3 (n = 22) | **0.65** | **0.001** | **- 0.603** | **0.003** |
| G1 (n = 135) | 0.071 | 0.410 | **- 0.247** | **0.004** |
| G2 (n = 29) | - 0.148 | 0.444 | **- 0.491** | **0.007** |
| G3-5 (n = 20) | 0.073 | 0.760 | **- 0.517** | **0.019** |

Note: ACR, albumin–creatinine ratio; eGFR, estimated glomerular filtration rate; DKD, diabetic kidney disease; G, GFR category (G1-G5); A, Albuminuria category (A1-A3);

**Table Supplement 2. The correlation of serum UA with urinary ACR and eGFR in adult-onset T1DM patients with good glycemic control**

| Classification | Spearman’s correlation analysis | | | |
| --- | --- | --- | --- | --- |
|  | Urinary ACR | | eGFR | |
|  | r | *P* - value | r | *P* - value |
| HbA1c < 7.0% (n = 35) | 0.308 | 0.072 | - 0.506 | 0.002 |

**Note**: ACR, albumin–creatinine ratio; eGFR, estimated glomerular filtration rate


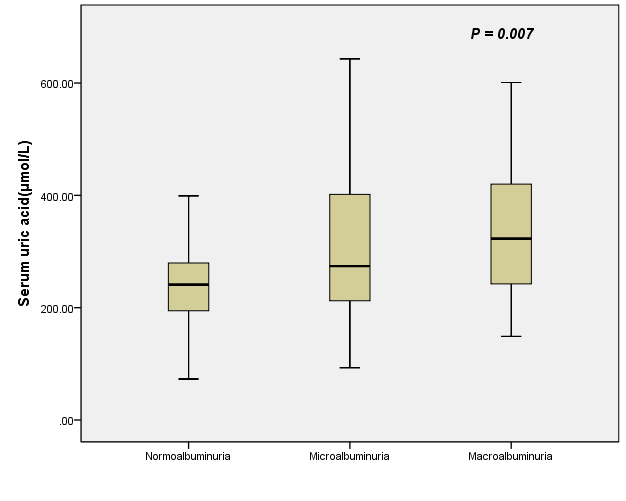


FIGURE Supplement 1: Serum UA in groups of albuminuria (the data of Figure 1 without those outliers). Data represent median (*P*25, *P*75). Participants were categorized as normoalbuminuria (ACR<30 mg/g), microalbuminuria (ACR 30–299 mg/g), and macroalbuminuria (ACR >300 mg/g).


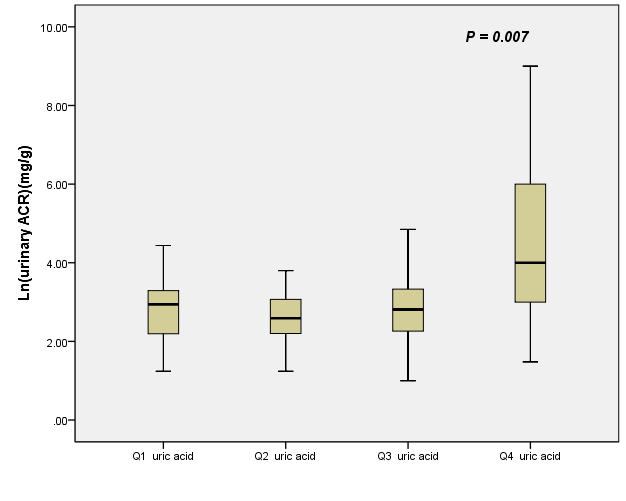


FIGURE Supplement 2：Urinary ACR in gender-specific quartiles of serum uric acid. Data represent the median (P25, P75). (The data of Figure 3 converted by natural logarithm and without those outliers)


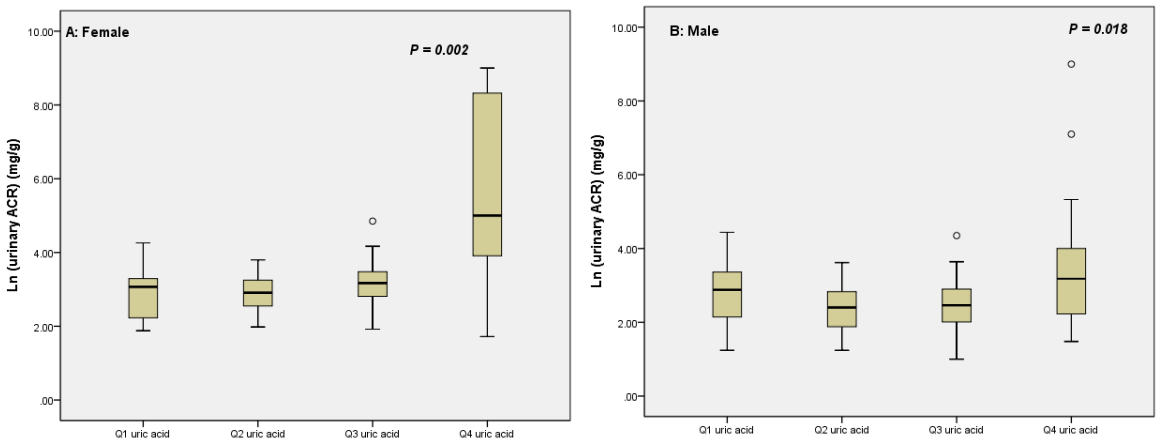


FIGURE Supplement 3：Urinary ACR in quartiles of serum uric acid (A: Female, B: Male). Data represent the median (P25, P75).


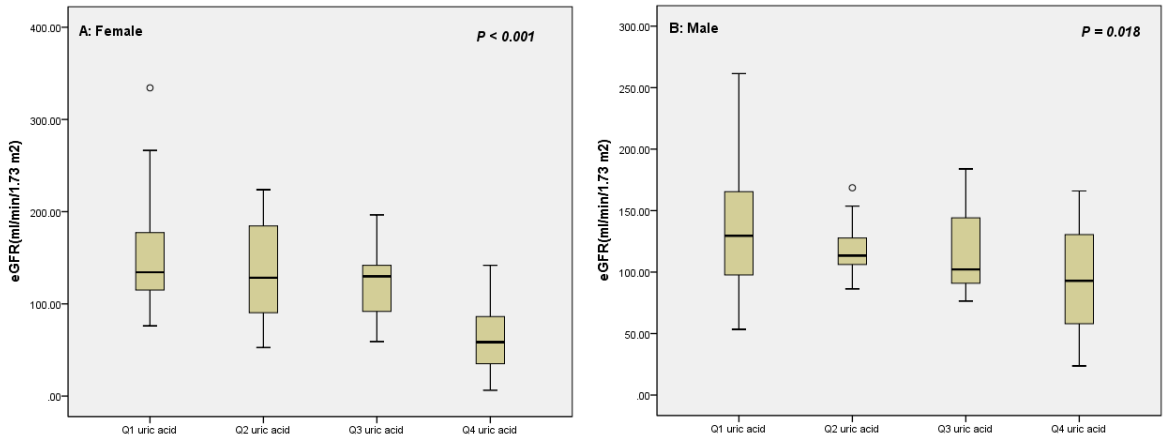


FIGURE Supplement 4: eGFR in quartiles of serum uric acid (A: Female, B: Male). Data represent the median (*P*25, *P*75).
